# Network toxicology, molecular docking and *in vitro* validation reveal the molecular mechanisms of bisphenol A-induced MASLD

**DOI:** 10.3389/fphar.2026.1905217

**Published:** 2026-10-30

**Authors:** Yue Li, Zhuo Xiong, Yundi Zhang, Chengyong Tang

**Affiliations:** 1 Phase I Clinical Trial Center, Bishan Hospital of Chongqing Medical University, Bishan Hospital of Chongqing, Chongqing, China; 2 Binzhou Institute of Health Technology, Binzhou, Shandong, China

**Keywords:** bisphenol A, hepatotoxicity, MASLD, molecular docking, network toxicology

## Abstract

**Background:**

Bisphenol A (BPA), a ubiquitous endocrine-disrupting chemical, is associated with metabolic dysfunction-associated steatotic liver disease (MASLD), yet its underlying molecular mechanisms remain elusive.

**Methods:**

This study combined network toxicology and molecular docking to explore BPA-induced hepatotoxicity targets and pathways in MASLD. BPA and MASLD-related targets were screened from multiple databases, with 886 and 3109 targets identified respectively; their intersection targets were analyzed via protein-protein interaction (PPI) network, and core targets were screened using Cytoscape Molecular Complex Detection (MCODE). The GSE163211 dataset was used for external validation of core genes, followed by Gene Ontology (GO) and Kyoto Encyclopedia of Genes and Genomes (KEGG) enrichment analyses, and molecular docking verified the binding affinity between BPA and key core targets.

**Results:**

We obtained 451 intersection targets, with 16 core targets (e.g., *JUN, IL6, TNF, TLR4*) identified; Gene Expression Omnibus (GEO) validation confirmed significantly upregulated expression of several core targets in MASLD samples (*P* < 0.05). GO enrichment revealed involvement in transcriptional regulation, protein binding and intracellular structural processes, while KEGG enrichment highlighted the PI3K-Akt, FoxO and cellular senescence pathways. Molecular docking showed BPA bound strongly to six key core targets with binding energies ranging from -5.3 to -8.2 kcal/mol.

**Conclusion:**

This study identifies the core targets and signaling pathways mediating BPA-induced MASLD, establishing an integrated computational framework for risk assessment of environmental hepatotoxicants and providing a theoretical basis for BPA regulatory policy formulation and MASLD mechanistic research.

## 1 Introduction

Bisphenol A (BPA; 2,2-bis (4-hydroxyphenyl) propane) is one of the most abundantly produced synthetic chemicals globally. It serves as a key feedstock for polycarbonate plastics and epoxy resins, and is widely distributed across a diverse range of consumer and industrial products in daily life, such as food containers, thermal paper receipts, water pipelines, toys, medical devices, and electronic appliances (Vandenberg et al., 2007). Owing to its massive production and extensive application, BPA is ubiquitously distributed in environmental compartments including air, riverine and marine waters, and soil (Yamazaki et al., 2015). Human exposure to BPA occurs routinely in daily life, primarily via oral ingestion and dermal contact. Structurally analogous to endogenous estrogen, BPA has been shown to trigger diverse estrogenic endocrine responses in biological systems both *in vitro* and *in vivo*. As a prototypical endocrine-disrupting chemical (EDC), it possesses marked estrogenic activity and strong binding affinity toward estrogen receptors (ERs) (Kang et al., 2007). Previous studies have shown that BPA is commonly present in human serum, urine, placental tissue, umbilical cord blood and breast milk (Geens et al., 2012; Rochester, 2013). In both humans and rodent models, gestational and lactational exposure to BPA at concentrations ≤50 μg/kg body weight per day has been linked to a spectrum of adverse health outcomes, including congenital anomalies, developmental and reproductive impairments, diabetes, obesity, cardiovascular disorders, chronic renal and respiratory diseases, and cancer (Rochester, 2013; Rezg et al., 2014). Clinical studies have shown that BPA levels in serum or urine are positively correlated with the increased risk of cardiovascular or cardiometabolic syndrome. BPA also is a risk factor for obesity, hypertension, heart and kidney diseases, such as acute kidney injury, chronic kidney disease, myocardial infarction, and stroke (Kang et al., 2023; Sonavane and Gassman, 2019).

As the primary detoxification organ in the human body, the liver is particularly susceptible to the adverse effects of EDCs. Epidemiological investigations from the United States (n = 7,605) (Kim et al., 2019) and (n = 944) (Verstraete et al., 2018) have demonstrated a positive association between urinary BPA concentrations and the risk of metabolic dysfunction-associated steatotic liver disease (MASLD). Moreover, elevated urinary BPA levels were positively correlated with increased serum concentrations of hepatic enzymes—including aspartate aminotransferase (AST), alanine aminotransferase (ALT), and gamma glutamyl-transferase (GGT)—as well as with the presence of hepatic dysfunction (Peng et al., 2023). BPA can also interact with the aryl hydrocarbon receptor (AhR), thereby modulating xenobiotic metabolism as well as steroid biosynthesis and metabolism (Bonefeld-Jorgensen et al., 2007; Zhuang et al., 2023). BPA exerts regulatory effects on a wide range of transcription factors. Numerous studies have verified that BPA promotes obesity by modulating adipogenic transcription factors including peroxisome proliferator-activated receptors (PPARα/γ), C/EBPs and Nrf2. Notably, this obesogenic action also induces hepatic lipid accumulation (Garcia-Arevalo et al., 2014; Biasiotto et al., 2016). Beyond its well-established obesogenic role, BPA structurally mimics endogenous estrogens, enabling it to bind to and modulate estrogen receptors (ERα/β). These receptors are highly expressed in hepatocytes, where they orchestrate critical processes including hepatic lipid homeostasis, glucose metabolism, and inflammatory signaling. BPA-mediated disruption of ER signaling cascades perturbs this delicate regulatory balance, promoting aberrant *de novo* lipogenesis, impairing mitochondrial fatty acid β-oxidation, and driving excessive triglyceride (TG) accumulation in hepatocytes. Furthermore, BPA interferes with additional nuclear receptors essential for lipid metabolism—specifically PPARα/γ (Ismael et al., 2022) and liver X receptors (LXRα/β)—thereby dysregulating hepatic lipid profiles and exacerbating the pathogenesis of MASLD (He et al., 2023).

MASLD is a heterogeneous spectrum of progressive hepatic diseases, covering a disease continuum from simple steatosis and metabolic dysfunction-associated steatohepatitis (MASH) to hepatic fibrosis, cirrhosis and ultimately hepatocellular carcinoma (HCC). Currently, it has become the predominant cause of chronic liver disease across the globe, affecting roughly 33% of the general population worldwide (Eslam et al., 2020; Rinella et al., 2023). MASLD has a multifactorial pathogenesis, which encompasses a cascade of pathological events: dysregulated lipid metabolism, inflammation, oxidative stress, and gut microbiota dysbiosis. Cumulative evidence has established a clear link between BPA exposure and the onset and progression of MASLD (Peng et al., 2023). Nevertheless, the exact molecular mechanisms responsible for BPA-triggered MASLD have not been fully elucidated. Considering the intricate nature of MASLD pathogenesis, which is modulated by diverse genetic, metabolic and environmental cues, BPA-induced hepatotoxicity cannot be attributed to any solitary molecular event or signaling pathway. While traditional experimental strategies have provided meaningful insights into discrete molecular interactions, they are inadequate to characterize the system-wide, multi-target and interconnected regulatory networks perturbed by environmental toxicants like BPA. A thorough mechanistic investigation therefore necessitates a systems-level perspective, which enables the integration of massive biological datasets to uncover key molecular nodes and functional pathways in these complex networks.

Currently, prominent methodological limitations still exist in exploring the regulatory effects of BPA on MASLD progression and identifying the specific molecular targets and signaling pathways mediating BPA-induced hepatic injury. To compensate for these research deficiencies, this study integrates network toxicology and molecular docking techniques to screen and characterize the core targets responsible for BPA-triggered hepatotoxicity. We conducted cross-database screening by integrating compound target and disease target databases, combined with protein-protein interaction (PPI) network analysis and validation based on Gene Expression Omnibus (GEO) datasets, so as to identify overlapping targets closely correlated with BPA toxicity and MASLD pathogenesis. Subsequent functional enrichment analysis was performed to elucidate the biological functions and core signaling pathways of these key targets. Molecular docking analysis was further adopted to verify the binding affinity between BPA and its pivotal targets. Finally, real-time quantitative PCR assay was utilized to detect the gene expression levels of targets with strong binding capacity to BPA. This comprehensive methodological framework provides a reliable computational strategy for the rapid risk assessment and priority classification of environmental hepatotoxicants. Furthermore, this study not only offers a feasible research paradigm for the toxicological evaluation of environmental pollutants, but also lays a solid theoretical foundation for the formulation of targeted regulatory policies and scientific risk management of BPA.

## 2 Materials and methods

### 2.1 Data sources and databases

The structural formula of BPA was retrieved from the PubChem database and downloaded the SMILES format. The SMILES format of BPA was input into the CTD database (Interaction Count ≥20), Swiss Target Prediction, GeneCards (Relevance score ≥20), and OMIM database to search for BPA targets. The targets retrieved from the four databases were summarized, and 193 duplicate items were removed. Finally, 886 BPA targets were obtained.

Through the combined screening of multiple databases, MASLD related targets were identified. Using the GeneCards database, 3,084 potential MASLD targets were obtained by setting (Relevance score ≥1); 49 related targets were obtained through the TTD database; 29 targets were retrieved from the OMIM database through the gene map module by searching for MASLD; 15 related targets were obtained by using the interaction module of the drug bank database; and 3 MASLD-related gene targets were obtained by screening from the PharmGKB database. After removing 71 duplicate items, a total of 3,109 gene targets were finally obtained.

### 2.2 Intersection target screening

The Venny tool in the SRplot (Tang et al., 2023) online data analysis platform was used to perform intersection analysis of BPA-related targets and MASLD-related targets, so as to obtain potential key targets of BPA-induced MASLD, and a Venn diagram was plotted.

### 2.3 Construction of PPI network and screening of core targets

The official STRING website (https://cn.string-db.org/) was accessed, and the “Multiple proteins” button was selected. The intersection targets were sorted into a list format and input into the target gene list, with the species set to “*Homo sapiens*”, followed by clicking the “search” button. The Network Type was set to “full STRING network”, the highest confidence score in Required score was set to 0.900, the FDR stringency was set to high, and unconnected nodes were hidden. The PPI network data was obtained and exported in TSV format.

The TSV file obtained in 2.3 was imported into Cytoscape_v3.10.3. All imported targets were scored using the Molecular Complex Detection (MCODE) tool, the group with the highest score was selected, and a network was generated according to the correlation degree of nodes.

### 2.4 External validation of GEO database

By searching for “MASLD” in the GEO public database, the species was selected as “*H. sapiens*”, and the Study type was set as “expression profiling by array”. The GSE163211 dataset was chosen for analysis. The dataset was standardized using R software, and the probe names in the dataset were converted to gene names of the GPL29503 (Nano String Counter) platform using the “org.Hs.eg.db” toolkit to obtain the expression data files of the sample probes corresponding to the genes. The expression data of the core target genes were imported into GraphPad Prism 8.0.1 for a Student’s t-test (*P < 0.05 was considered significant).

### 2.5 Enrichment analyses for GO terms and KEGG pathways

The intersection targets were imported into the DAVID database, with the gene type set as “OFFICIAL_GENE_SYMBOL”, the species type as “*H. sapiens”*, and the List type as “gene list”. The list was submitted, and the “Functional Annotation Chart” button was clicked to view the results. The GOTERM BP DIRECT, GOTERM CC DIRECT, and GOTERM MF DIRECT result data were exported separately, sorted in descending order by P-value, and the top 20 terms were selected. The P values were processed by negative logarithm, and the gene ratio, count, and log_10_(P value) of the top 20 Terms were imported to the online analysis platform for visualization.

### 2.6 Molecular docking

The three-dimensional (3D) crystal structures of core target proteins were downloaded from the Protein Data Bank (PDB, https://www.rcsb.org/) with the criteria of resolution <2.5 Å, human-derived, and no mutation. The SDF format file of the target compound was downloaded from the PubChem database. In this study, molecular docking simulation was performed using the CB-Dock2 platform (Liu et al., 2022), which conducts ligand/protein preprocessing for the imported protein structures. Protein preprocessing: PDB format protein files were read, missing side-chain atoms and hydrogen atoms were automatically supplemented, crystal water and heteroatoms were removed, and missing residues were labeled, providing a high-quality structural basis for subsequent cavity detection and docking. Ligand preprocessing: RDKit was used to generate initial 3D conformations, hydrogen atoms were added, and partial charges were calculated to meet the requirements of docking energy calculation. AutoDock Vina (1.2.0 version) was invoked for molecular docking, and binding conformations and Vina scores (kcal/mol) were calculated based on energy terms such as intermolecular hydrogen bonds, hydrophobic interactions, and electrostatic interactions. The Vina score is the core quantitative index of CB-Dock2 docking results, which is essentially an approximation of the binding free energy. A lower score (larger absolute value of negative value) indicates a stronger binding affinity between the ligand and the protein.

### 2.7 Cell culture

HepG2 cells were purchased from the Cell Bank of the Chinese Academy of Sciences. The cells were cultured in culture flasks containing Dulbecco’s modified Eagle medium (DMEM) containing 10% fetal bovine serum and placed in a cell incubator containing 5% CO_2_ at 37 °C ± 2 °C, and the medium was changed periodically.

### 2.8 BPA preparation

BPA (Macklin B802575-25g) molecular weight: 228.28 g/mol, raw material purity: 99%. A 100 mM BPA stock was prepared in DMSO (0.22 μm filtered) and serially diluted with DMSO to obtain 10 and 100 μM intermediate solutions. These were diluted in serum-free complete medium to final BPA concentrations of 10, 100 nM and 1, 10, 100 μM. All groups contained an equal DMSO final concentration of 0.05%.

### 2.9 Real-time quantitative PCR assay

After HepG2 cells were treated with 10 μM BPA for 48 h, total cellular RNA was isolated using RNA Extraction buffer (Yeasen). RNA concentration and purity were measured by NanoDrop 2000 spectrophotometer. One microgram of total RNA was reverse-transcribed into cDNA using Hifair® AdvanceFast 1st Strand cDNA Synthesis Kit (No Dye) (Yeasen, Cat.No. 11150ES60). qRT-PCR amplification was performed on a CFX96 Real-Time PCR detection system (Bio-Rad, United States) using Hieff® qPCR SYBR Green Master Mix (Yeasen, Cat.No. 11201ES08). The thermal cycling conditions were as follows: initial denaturation at 95 °C for 3 min; 40 cycles of 95 °C for 10 s and 60 °C for 30 s. Melting-curve analysis was performed after amplification to confirm the specificity of amplified products. β-actin was used as the internal reference gene. Relative mRNA expression of target genes (*IL6*, *IFNG*, *JUN*, *TLR4*) was calculated with the 2^−ΔΔCt^ method. Three independent biological replicates were conducted, with three technical replicates for each sample.

### 2.10 Lipid droplet staining

Intracellular lipid accumulation was assessed by Nile Red and Oil Red O staining. Cells were washed twice with PBS before fixation. For Nile Red staining, cells were fixed with 4% paraformaldehyde for 15 min, incubated with Nile Red and Hoechst 33342 (1:1,000) at 37 °C in the dark for 20 min. Images were acquired on an inverted fluorescence microscope with identical acquisition settings, using the TRITC channel for Nile red and the DAPI channel for Hoechst 33342 (scale bar = 200 μm). For Oil Red O staining, fixed cells were rinsed with 60% isopropanol for 5 min and stained with freshly prepared Oil Red O working solution for 20 min at room temperature. After rinsing with 60% isopropanol to remove unbound dye and washing with distilled water, cell nuclei were counterstained with hematoxylin for 2 min. Bright-field images were acquired after a final wash with distilled water (scale bar = 200 μm).

## 3 Results

### 3.1 BPA targets and MASLD targets

By searching four databases for BPA targets, 261 targets with Interaction Count ≥20 were obtained from the CTD database, 47 from Swiss Target Prediction, 198 from OMIM, and 573 from GeneCards with Relevance score ≥20. Summarizing the targets retrieved from the four databases, 193 duplicate items were removed, and finally 886 BPA targets were obtained (Figure 1).

**FIGURE 1:**
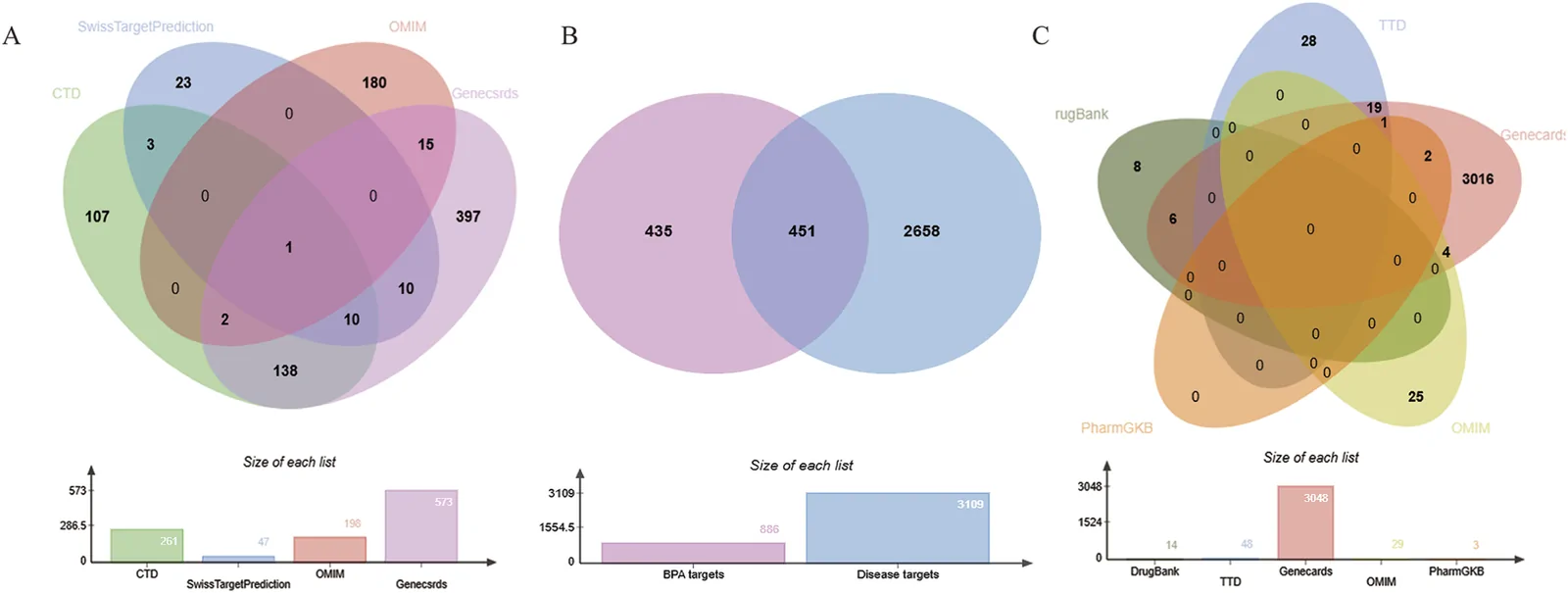
Venn diagram of BPA targets, MASLD targets and their intersection targets: **(A)** Venn diagram of BPA targets; **(B)** Venn diagram of the intersection of BPA and MASLD targets; **(C)** Venn diagram of MASLD disease targets.

### 3.2 Analysis of PPI interaction network of intersection targets

The 451 common BPA-MASLD intersection targets were imported into the STRING database (version 12.5, species: *Homo sapiens*). The confidence threshold was set to a combined score >0.9 (highest confidence). Isolated nodes without interactions were hidden. The maximum number of first-shell interactors was restricted to no more than 50, while second-shell interactors were disabled. All available active interaction sources, including Textmining, Experiments, Databases, Co-expression, Neighborhood, Gene Fusion, Co-occurrence, and Evidence transfer, were enabled. The PPI network data was exported in TSV format. The PPI analysis yielded a network containing 493 nodes and 3,453 edges, with an average local clustering coefficient of 0.46.

### 3.3 Core target network

The PPI data was imported into Cytoscape_v3.10.3 for visualization analysis. The MCODE plugin was used for core module screening using the plugin’s default parameters: Degree Cutoff = 2, K-Core = 2, Node Score Cutoff = 0.2, Max Depth = 100, Haircut enabled, Fluff disabled, Node Density Cutoff = 0.1, and “In Whole Network” was selected. The MCODE tool calculates the node score, with a higher score indicating a denser local network and a more core potential function. By selecting the group with the highest score (10), the number of nodes was 16, and the number of connections was 75. The core nodes were: JUN, IL6, TNF, SMAD3, TLR4, IL1B, IFNG, CXCL8, FOS, IL10, CCL2, CCL5, CXCL10, IL17A, IL13, IL4 (Figure 2).

**FIGURE 2:**
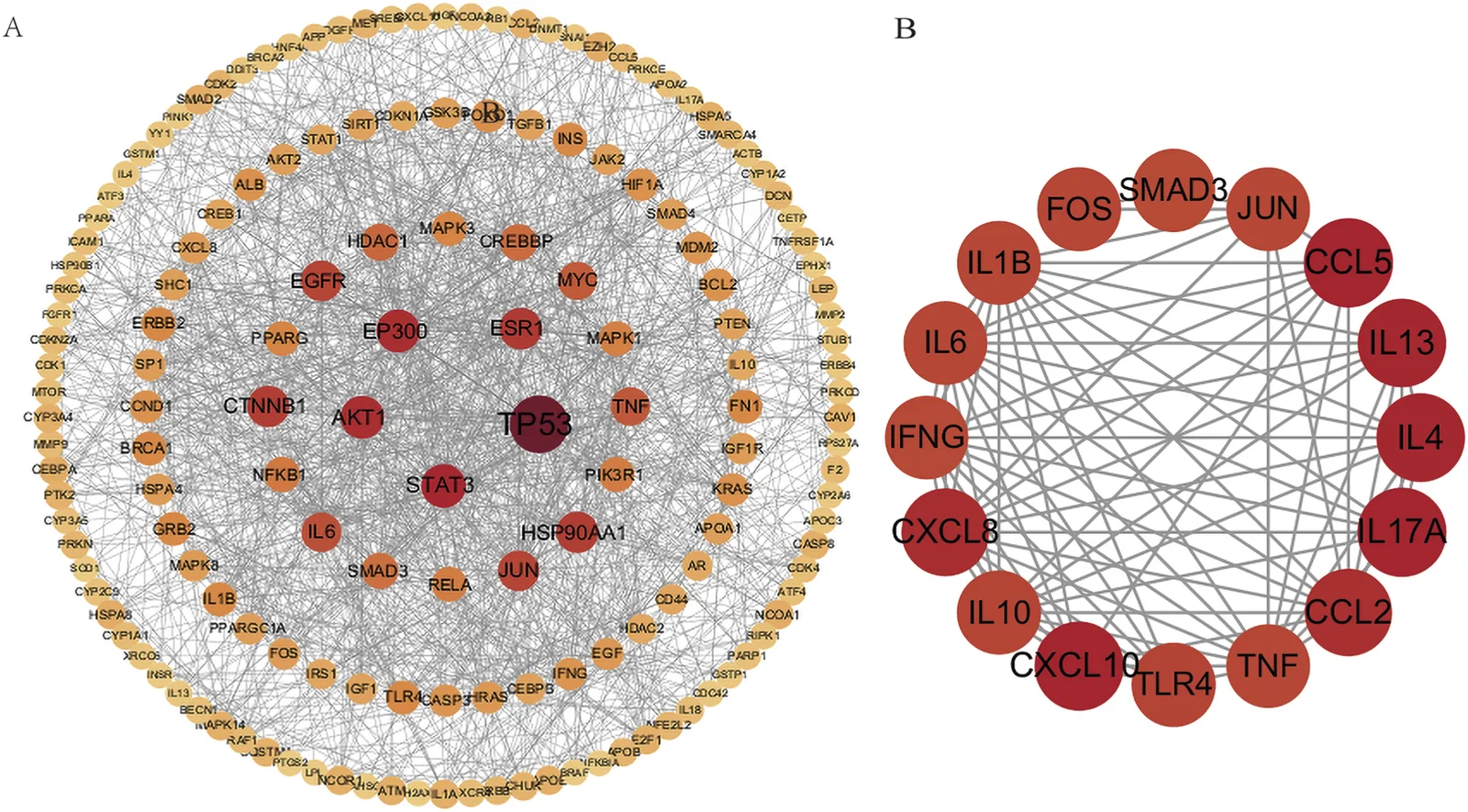
Protein interaction network and core gene interaction network of BPA-induced MASLD. **(A)** The interaction network of the intersection targets of BPA and MASLD with degree ≥10; **(B)** The interaction network of the core targets with the highest MCODE score. The larger and darker the node, the higher the score; the lines between nodes represent the interaction relationship between genes.

### 3.4 Validation of core genes

The GSE163211 dataset was randomly selected to verify the expression profiles of core genes. This dataset included 76 samples with normal liver biopsy results as the control group and 242 patients diagnosed with MASLD by liver biopsy as the disease group. R language was used to extract the gene expression matrix, and Student’s t-test was performed to analyze the differences in the expression of core genes between the two groups. Since no corresponding probes could be mapped to these three transcripts (*FOS, IL10, IL13*) using the GPL29503 platform, their expression data could not be extracted for differential expression analysis. For this reason, only 13 core genes with valid probe information are presented in Figure 3. The results showed that the expression levels of some core genes were significantly different (*P* < 0.05), and the expression levels in the disease group were higher than those in the normal group.

**FIGURE 3:**
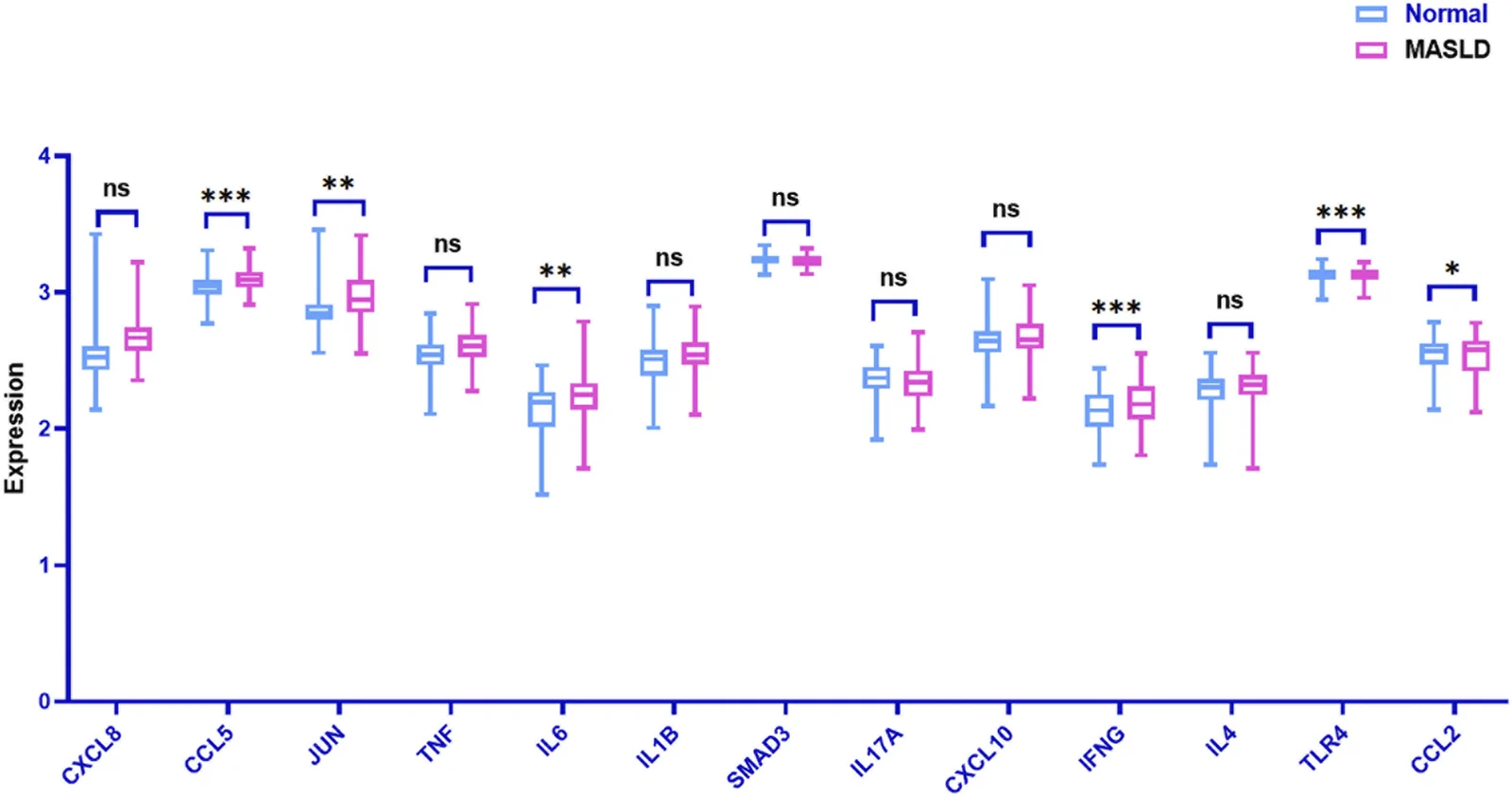
Differential expression profiles of 13 core genes in a random dataset. Blue: control group; Pink: MASLD group. *P < 0.05; **P < 0.01; ***P < 0.001; ns, there was no significant difference.

### 3.5 Enrichment analysis of gene ontology terms and KEGG pathways

To clarify the biological characteristics of the identified core targets, GO enrichment analysis was performed on 451 intersection targets of BPA and MASLD using the “cluster Profiler” package in R language. This analysis was divided into three functional domains: biological process (BP), cellular component (CC), and molecular function (MF). The significance of enrichment was quantified using the adjusted *P*-value converted in -log_10_ units (the larger the value, the more significant the enrichment).

As shown in Figures 4–6, GO-BP was associated with transcriptional regulation, cell proliferation, and signaling pathway processes. The cellular components involved in GO-CC included cytoplasm, nucleus, nucleoplasm, cytosol, and intracellular organelles. GO-MF was related to molecular functions such as protein binding, DNA binding, transcription factor binding, and enzyme binding.

**FIGURE 4:**
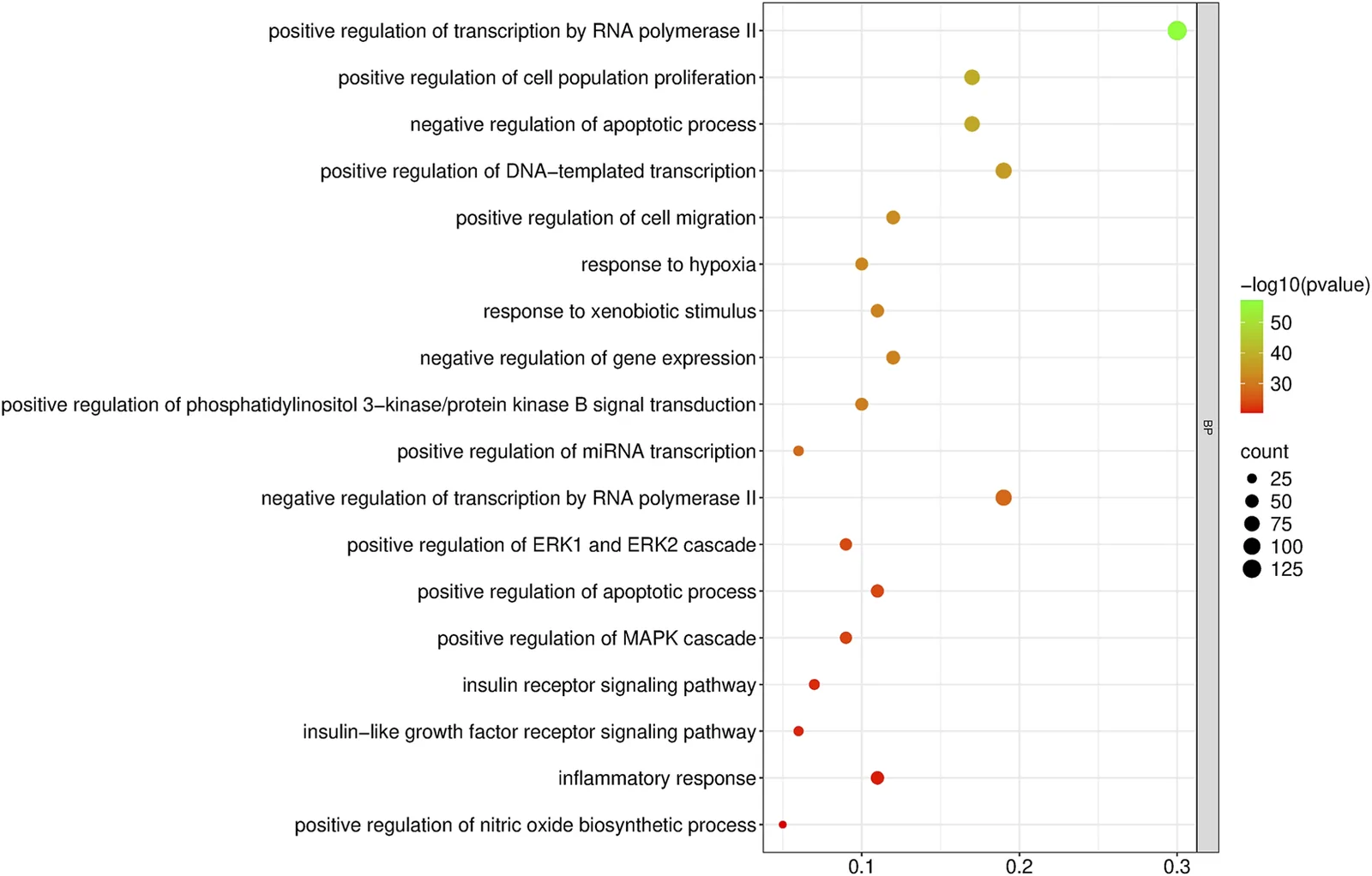
GO-BP enrichment dot plots of BPA-MASLD intersection targets. Note: dot color = −log_10_(adjusted P-value) (darker = more significant); dot size = target count per term.

**FIGURE 5:**
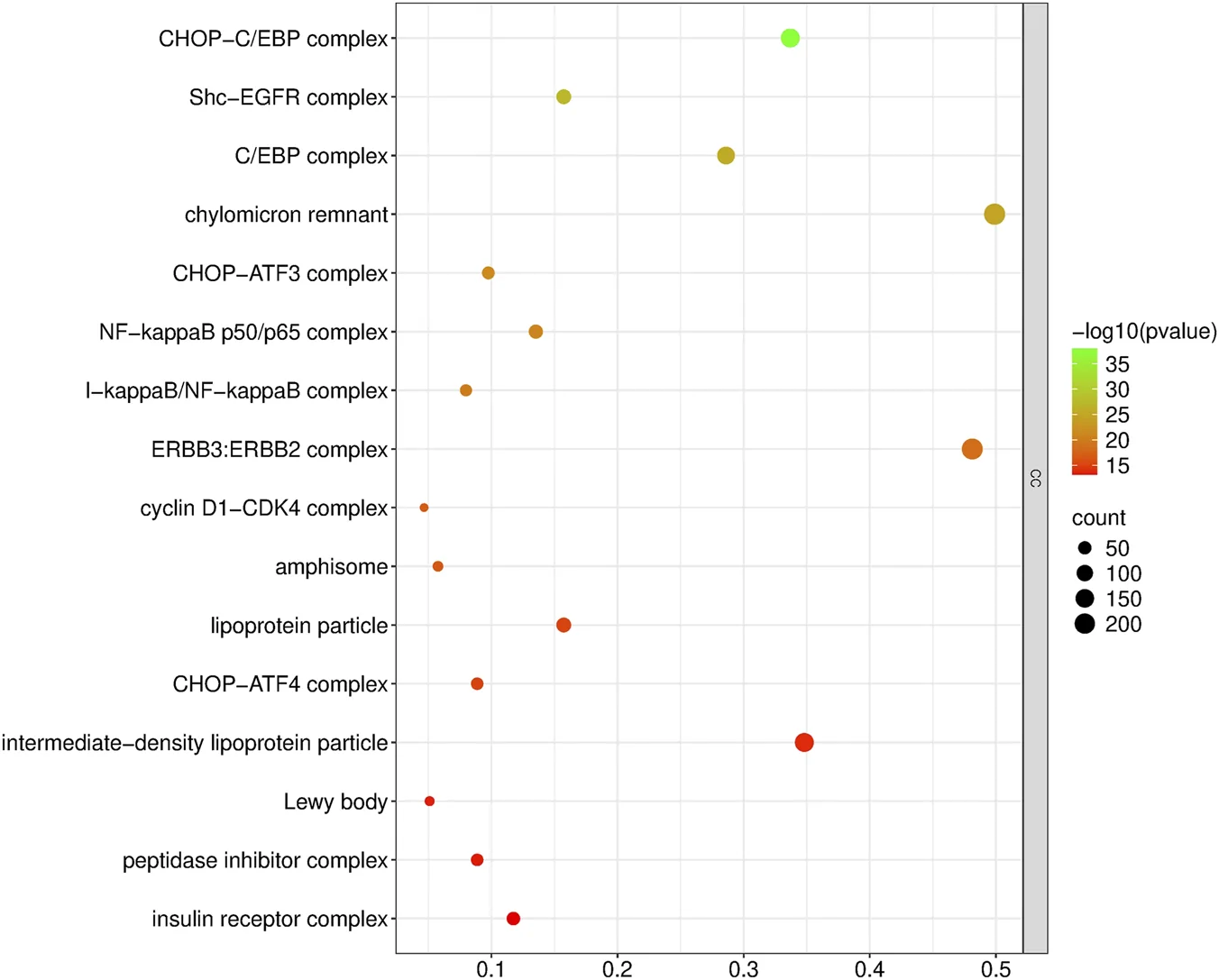
GO-CC enrichment dot plots of BPA-MASLD intersection targets.

**FIGURE 6:**
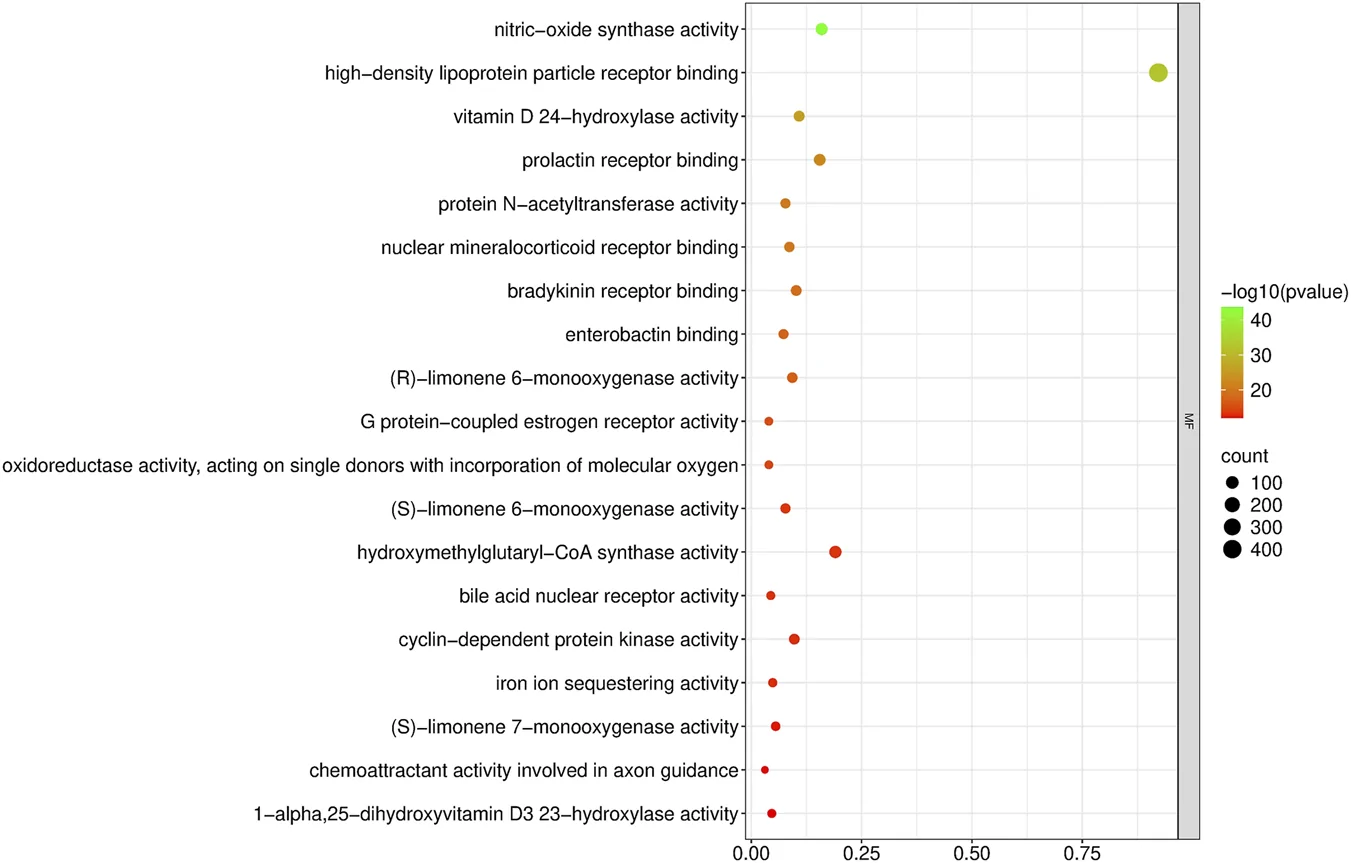
GO-MF enrichment dot plots of BPA-MASLD intersection targets.

KEGG pathway enrichment analysis identified 200 pathways (*P* < 0.05), mainly including the FoxO signaling pathway (Count = 45), PI3K-Akt signaling pathway (Count = 72), and cellular senescence pathway (Count = 44). It was also associated with disease-related pathways such as cancer, lipid and atherosclerosis, various cancer subtype pathways, infection-related pathways, and the AGE-RAGE signaling pathway in diabetic complications. As shown in Figure 7, the top 20 KEGG pathways were visualized using bar charts.

**FIGURE 7:**
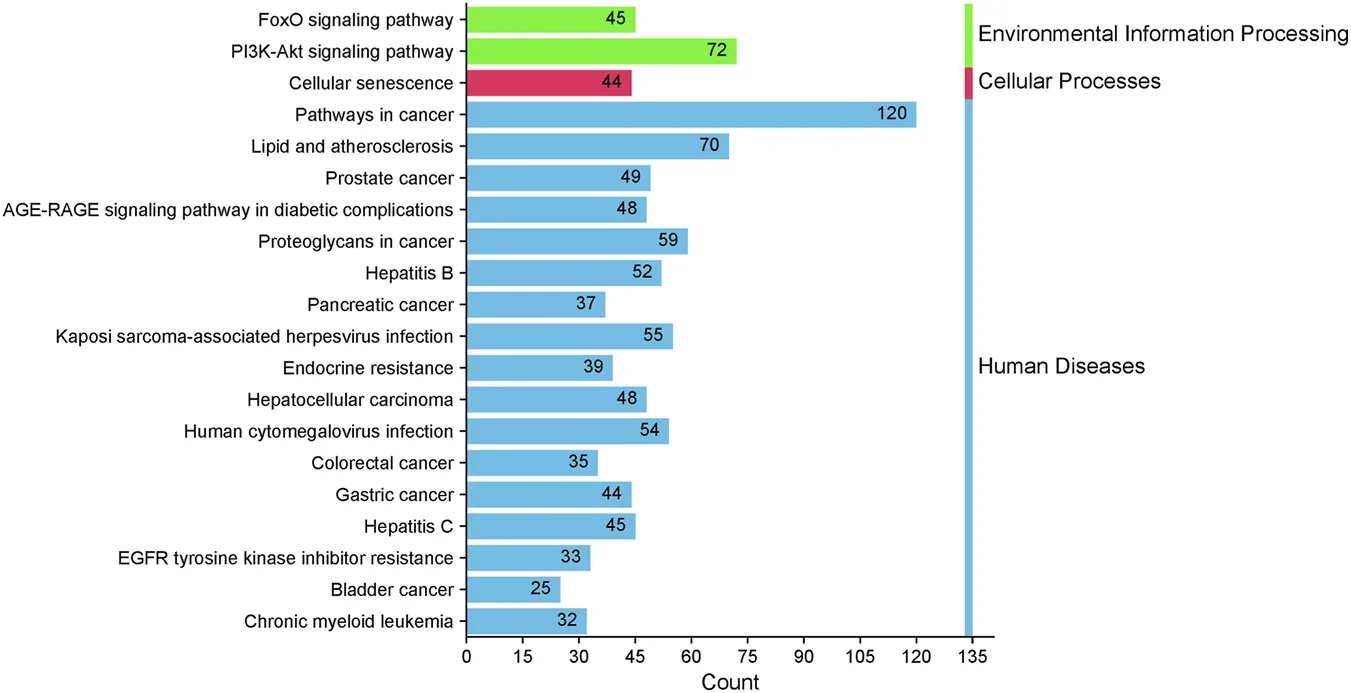
Top 20 KEGG enrichment bar plots of BPA-MASLD intersection targets. Note: Bar length = target count; color = −log_10_(adjusted P-value); pathways grouped by functional categories (right).

### 3.6 Molecular docking study on core targets of BPA and MASLD

The PPI network with the highest score, containing 16 protein molecules, was screened using the MCODE algorithm in Cytoscape. Six core targets (JUN, IL6, TLR4, IFNG, CCL2, CCL5) were verified to have a significant correlation with MASLD through the GSE163211 dataset in the GEO database. To explore potential molecular recognition events, molecular docking analysis was performed on these core targets to investigate the interactions between BPA and the six core targets. The docking results obtained by CB-Dock2 (Liu et al., 2022) showed that the binding energies between BPA and the target proteins ranged from −5.3 to −8.2 kcal/mol. Generally, a binding energy <0 indicates binding activity, while a value lower than −5.0 kcal/mol indicates strong binding activity, and a lower value indicates a stronger interaction. The results demonstrated that BPA had a strong affinity with the six core targets, suggesting spontaneous binding and implying their key roles in the molecular mechanism of BPA-induced MASLD. These computational results clearly indicated (Table 1) that BPA had a strong binding affinity with all six prioritized core targets. The detailed three-dimensional (3D) binding conformations and interaction modes of each BPA core target complex calculated by CB-Dock2 were fully visualized and presented in Figure 8. The 2D images showed the docking diagrams with the receptor framework hidden to visualize the docking content and precise localization more clearly.

**TABLE 1:** The result of molecular docking.

Target proteins	PDB ID	Vina score (kcal/mol)	Cavity volume (Å3)	Key interacting residues
CCL5	6STK	−6	28	Chain A: ALA12 ALA13 ALA14 ILE15 ALA16 TYR29 THR30 SER31 GLY32 SER35
CCL2	3IFD	−5.3	43	Chain A: CYS12 TYR13 ASN14 PHE15 ARG30 THR32 SER33 SER34 CYS36 PRO37
JUN	1JNM	−8.2	3,333	Chain A: ARG270 LYS273 LEU274; Chain B: ALA266 SER267 LYS268 SER269
TLR4	3FXI	−7.5	2,669	Chain B: GLU439 PHE440 SER441 LEU444 PHE463; Chain C: VAL24 CYS25 ILE32 ILE44
IFNG	1FYH	−7.5	1,094	Chain A: PHE29 GLN48 ILE49 SER51 PHE52 LYS55 LEU95 THR96 GLN248 ILE249
IL6	1ALU	−7.5	436	Chain A: GLU42 THR43 LYS46 SER47 ARG104 PHE105 GLU106 SER107 SER108 GLN156

**FIGURE 8:**
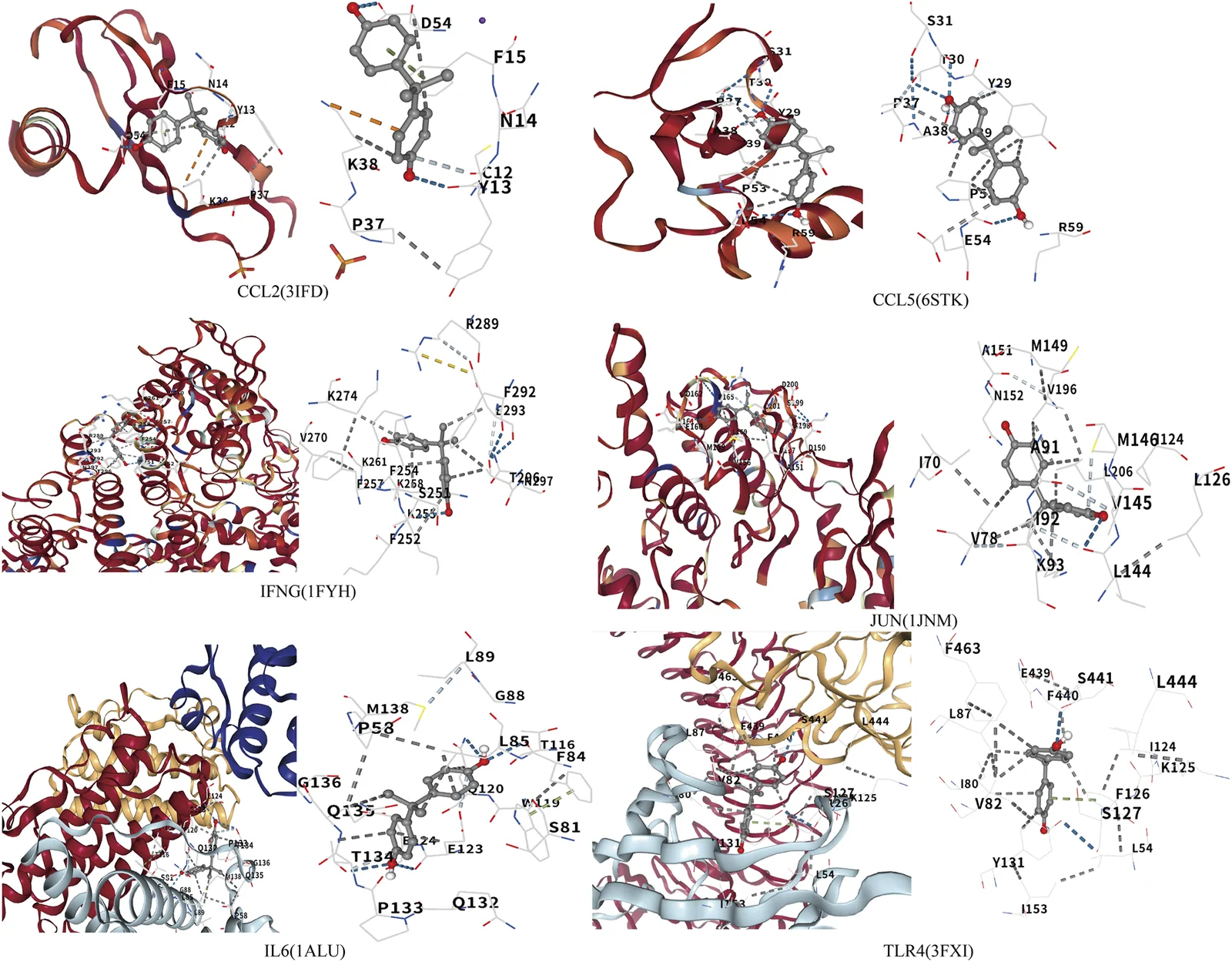
3D and 2D interaction diagrams of BPA docked with six core target proteins, including CCL2 (PDB: 3IFD), CCL5 (PDB: 6STK), IFNG (PDB: 1FYH), JUN (PDB: 1JNM), IL6 (PDB: 1ALU), and TLR4 (PDB: 3FXI).

### 3.7 BPA exposure induced lipid accumulation and upregulated core targets in HepG2 hepatocytes

To test whether BPA exposure could induce fatty liver, HepG2 cells were treated with 10 μM BPA to mimic the BPA-exposed environment. Time-gradient treatments (0 h/NC, 12, 24, 48 h) were performed to characterize the dynamic changes of lipid deposition. As shown in Figures 9A,B, BPA triggered progressive intracellular lipid accumulation in HepG2 cells over time. The Nile Red fluorescence intensity was markedly elevated in BPA-treated cells, and Oil Red O staining revealed gradually increased cytoplasmic red lipid droplets from 12 to 48 h, while few lipid droplets were observed in the NC group.

**FIGURE 9:**
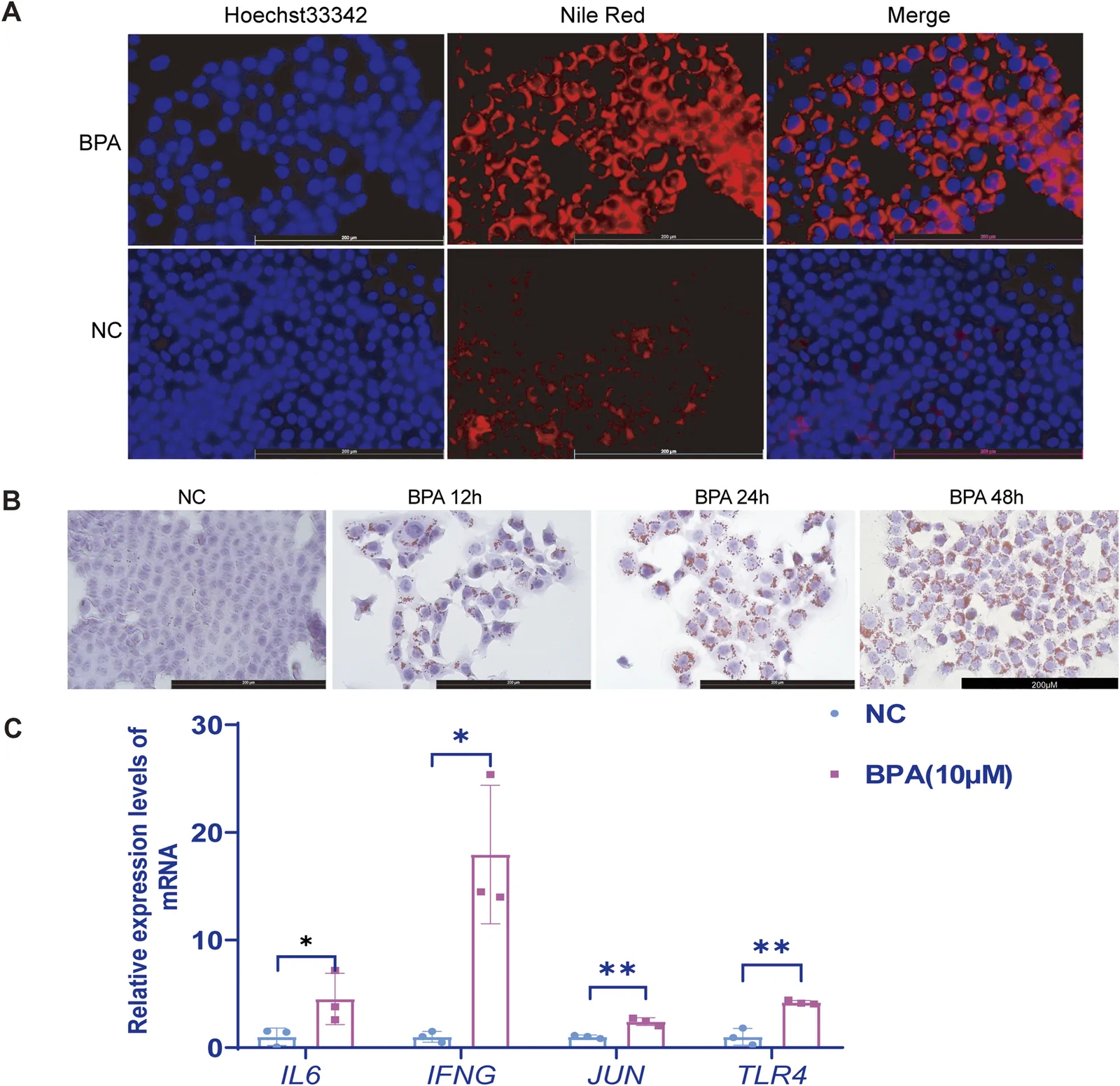
BPA exposure induced lipid accumulation and transcriptional upregulation of inflammation-related genes in HepG2. **(A)** Representative fluorescence images of Nile Red staining for intracellular lipid droplets in HepG2 cells with or without 10 μM BPA treatment. Nuclei were counterstained with Hoechst 33342. Scale bar = 200 μm; **(B)** Representative Oil Red O staining micrographs showing intracellular lipid deposition in HepG2 cells after 10 μM BPA exposure for 0 h (NC), 12, 24 and 48 h. Scale bar = 200 μm; **(C)** qRT-PCR analysis of relative mRNA expression levels of *IL6*, *IFNG*, *JUN* and *TLR4* in HepG2 cells following 48 h of 10 μM BPA stimulation. β-actin served as the internal reference gene. Data are presented as mean ± SD. ns, not significant; *P < 0.05, **P < 0.01.

Total RNA was extracted from HepG2 cells after 48 h of BPA stimulation, and qRT-PCR was performed to detect hub genes with molecular docking Vina scores < −7 kcal/mol. As shown in Figure 9C, the mRNA levels of *IL6* (*P* < 0.05), *IFNG* (*P* < 0.05), *JUN* (*P* < 0.01), and *TLR4* (*P* < 0.01) were significantly increased after BPA treatment. Collectively, these findings demonstrate that BPA exposure induces time-dependent lipid accumulation and activates the *TLR4*-associated inflammatory pathway in hepatocytes.

## 4 Discussion

MASLD has emerged as a major global public health challenge. It progresses sequentially from simple hepatic steatosis to MASH, liver fibrosis, and ultimately cirrhosis, thereby imposing a substantial burden on individual health and global healthcare system (Friedman et al., 2018). Its pathogenesis arises from intricate crosstalk among genetic predisposition, metabolic dysfunction, and environmental factors, among which EDC exposure serves as a critical environmental risk factor (Rezg et al., 2014). BPA, a typical EDC widely used in plastics, food packaging, and daily necessities, exhibits high environmental persistence and bioaccumulation (Rezg et al., 2014). Previous studies have confirmed that BPA exposure is associated with metabolic abnormalities such as insulin resistance and lipid metabolism disorders, which are closely linked to MASLD progression (Eslam et al., 2025). Although conventional experimental approaches provide valuable evidence for individual molecular interactions, they fall short of capturing the holistic, multi-target, and multi-pathway regulatory networks perturbed by environmental toxicants such as BPA. In-depth mechanistic exploration therefore requires a systems-level strategy capable of integrating multidimensional biological data to identify core nodal genes and pivotal signaling pathways within these complex regulatory networks. Such methodological limitations have hindered comprehensive elucidation of the precise molecular mechanisms underlying BPA-triggered MASLD pathogenesis.

To address the aforementioned knowledge deficit, this study integrated multi-source database screening, PPI network profiling, and molecular docking analysis to systematically dissect the molecular mechanisms by which BPA induces MASLD. In total, 886 putative BPA target genes were retrieved from four mainstream databases, including CTD, Swiss Target Prediction, OMIM, and Gene Cards. After intersecting with MASLD-associated genes, 451 overlapping targets were obtained and defined as the core regulatory molecules linking BPA exposure to MASLD occurrence. Further PPI network analysis yielded 493 nodes and 3,453 edges, with an average local clustering coefficient of 0.46, demonstrating a compact and functionally synergistic interaction network among these key genes. These results strongly support that BPA-mediated MASLD pathogenesis depends on the coordinated modulation of intricate target networks, instead of discrete individual molecular interactions.

Core targets module analysis was performed using the MCODE plugin in Cytoscape, yielding 16 hub targets with the optimal clustering score (Score = 10), namely, *JUN, IL6, TNF, SMAD3, TLR4, IL1B, IFNG, CXCL8, FOS, IL10, CCL2, CCL5, CXCL10, IL17A, IL13*, and *IL4*. GEO dataset validation (GSE163211) revealed that six key hub genes (*JUN*, *IL6*, *TLR4*, *IFNG*, *CCL2*, *CCL5*) were markedly overexpressed in MASLD patients compared with healthy individuals (*P* < 0.05), strongly supporting their critical regulatory roles in MASLD progression. Nevertheless, the remaining core targets showed no significant differential expression in this dataset. Such inconsistent results are likely attributable to sample heterogeneity and discrepant pathological stages within the dataset. It is also plausible that these genes participate in BPA-mediated MASLD through subtle, context-dependent mechanisms, necessitating further systematic validation in future studies.

To further validate the underlying regulatory mechanism, we detected the mRNA expression of four pivotal genes with high binding affinity to BPA (Vina score < −7.0 kcal/mol). Consistent with our bioinformatic predictions, *JUN*, *TLR4*, *IL6*, and *IFNG* were markedly upregulated upon BPA treatment. These hub genes are well-established contributors to MASLD pathogenesis. Notably, *JUN* constitutes a core subunit of the AP-1 transcription complex, which governs the transcription of genes involved in hepatic lipid metabolism and inflammatory responses, and its excessive activation drives hepatocyte steatosis and fibrotic progression (Vogt, 2002). As a structural analog of 17β-estradiol, BPA targets and modulates hepatic ERs, which are essential for maintaining hepatic lipid metabolic homeostasis (Sommerauer et al., 2024; Palmisano et al., 2017). Beyond the classical ER-mediated genomic pathway, our identification of *TLR4* and *JUN* as key hub nodes highlights a previously underemphasized ER-independent mechanism of BPA hepatotoxicity. BPA directly sensitizes the TLR4/MD-2 complex and initiates sterile pro-inflammatory signaling cascades independent of bacterial endotoxin stimulation (Karunarathne et al., 2021). Subsequent *TLR4* activation induces kinase-mediated JUN phosphorylation, which is a prerequisite for AP-1 transcriptional activation. The interactive crosstalk between phosphorylated JUN and ER signaling plays a decisive role in hepatic metabolic regulation (Schulien et al., 2019); phosphorylated JUN can antagonize the protective effects of ER-α, leading to the transcriptional upregulation of lipogenic genes such as *SREBP-1c* and *FASN* (Jin et al., 2022). Consequently, the BPA-induced TLR4/JUN axis shifts the hepatic environment toward a pro-inflammatory and pro-steatotic state, accelerating the transition from simple steatosis to MASH.

Beyond this central axis, *IL6* is produced and secreted by a variety of cells, including macrophages, and is a key proinflammatory cytokine mediating chronic low-grade inflammation in MASLD (Mihara et al., 2012). As MASLD progresses, *IL6* levels increase, and IL6 protein content has been shown to be associated with the severity of MASLD (Tutunchi et al., 2021; Giraldez et al., 2021). TLR4 as a pattern recognition receptor (Peng et al., 2021), mediates lipopolysaccharide-induced hepatic inflammation and insulin resistance, which is a crucial link between gut microbiota dysbiosis and MASLD progression (Tanimura et al., 2008). Chemokines such as *CCL2* (Liu et al., 2021), *CCL5*, and *CXCL10* regulate the recruitment of inflammatory cells to the liver (Haukeland et al., 2006; Fuchs et al., 2022), exacerbating local inflammatory responses and promoting disease progression (Fuchs et al., 2022). *IFNG* is produced by lymphocytes activated by specific antigens or mitogens (Gray and Goeddel, 1982). IFN-γ mainly plays a pro-inflammatory and pro-fibrotic role in the progression of MASLD, driving the development from simple steatosis to MASH and liver fibrosis (Luo et al., 2013). *SMAD3* participates in the TGF-β signaling pathway (Yamaguchi et al., 2022), which is a core pathway driving hepatic fibrosis. Conversely, *IL10*, *IL4*, and *IL13* exhibit anti-inflammatory effects, suggesting a complex balance between pro-inflammatory and anti-inflammatory responses in BPA-induced MASLD, with BPA potentially disrupting this balance to favor pathological progression.

Functional enrichment analyses, including GO and KEGG analyses, were conducted to systematically decipher the biological functions of the intersecting targets linking BPA exposure and MASLD. GO-BP terms were mainly enriched in transcriptional regulation, cell proliferation, and signaling pathway processes, demonstrating that BPA could modulate the transcriptional activity of key genes related to hepatic metabolism and inflammation, thereby impairing normal liver physiological homeostasis. GO-CC analysis further confirmed the subcellular localization of these core genes in the cytoplasm, nucleus, and nucleoplasm, which matches the intrinsic distribution characteristics of transcription factors (*JUN*, *FOS*) and pro-inflammatory cytokines (*IL6*, *TNF*) and supports their potential functional crosstalk. Moreover, GO-MF enrichment centered on protein binding, DNA binding, and transcription factor binding, illustrating that BPA-induced hepatic dysfunction is closely attributed to the disrupted molecular binding events of core targets, which further dysregulates downstream signaling transduction cascades.

KEGG pathway analysis identified 200 significantly enriched pathways (*P* < 0.05), with key pathways including the PI3K-Akt signaling pathway (Count = 72), FoxO signaling pathway (Count = 45), cell senescence pathway (Count = 44), lipid and atherosclerosis, and AGE-RAGE signaling pathway in diabetic complications. The PI3K-Akt pathway is a core regulator of lipid metabolism and insulin signaling (Bu et al., 2024); its abnormal activation can promote hepatic lipid synthesis and accumulation, which is a key mechanism in MASLD development. The FoxO signaling pathway participates in the regulation of hepatic glucose and lipid metabolism (Sha et al., 2024), and its inhibition by BPA may exacerbate insulin resistance and steatosis. The enrichment of lipid and atherosclerosis pathway suggests that BPA-induced MASLD may be closely associated with systemic metabolic disorders, increasing the risk of cardiovascular complications. Additionally, the involvement of multiple cancer-related pathways indicates that long-term BPA exposure may promote the malignant transformation of MASLD, which is consistent with previous findings that MASLD is a risk factor for hepatocellular carcinoma (Kim et al., 2019).

Molecular docking analysis assessed the binding affinity between BPA and six key core targets (JUN, IL6, TLR4, IFNG, CCL2, CCL5), with binding energies ranging from −5.3 to −8.2 kcal/mol. Given that binding energies below −5.0 kcal/mol are generally considered indicative of favorable spontaneous binding, these results suggest that BPA may interact with these core targets with relatively high affinity, offering structural clues for potential regulatory effects of BPA on the above-mentioned molecules. The stable binding between BPA and core targets such as IL6, TLR4, and JUN further supports the hypothesis that BPA mediates MASLD through interfering with inflammatory signaling and transcriptional regulation.

Although this study systematically elucidates the potential mechanisms of BPA-mediated MASLD pathogenesis, several inherent limitations should be acknowledged. First, the current findings are merely derived from bioinformatic prediction, lacking rigorous *in vivo* and functional verification. The causal linkage between BPA exposure and dysregulated core targets and signaling pathways in MASLD remains to be validated by further cellular and animal experiments. Second, MASLD exhibits prominent pathological and phenotypic heterogeneity. Different MASLD subtypes may respond differentially to BPA toxicity, yet such subtype-specific effects were not considered in our analytical framework. Third, human environmental exposure to BPA rarely occurs independently but coexists with other prevalent EDCs, including phthalates and bisphenol S. The interactive synergistic or antagonistic effects of combined EDCs exposure on MASLD development remain unclarified. Fourth, in this study, 10 μM BPA was used for *in vitro* mechanistic investigation; this concentration exceeds typical human environmental exposure levels and cannot directly mimic chronic low-dose BPA exposure in humans, and further animal experiments are required to verify the cumulative effects of low-dose BPA.

In summary, this study comprehensively uncovered the core hub genes and key signaling cascades responsible for BPA-driven MASLD via integrated bioinformatic analysis. Functional interpretation revealed that BPA exacerbates MASLD progression through multiple pathogenic mechanisms, including disrupted transcriptional modulation, sustained chronic inflammation, and aberrant lipid and insulin metabolic signaling. The identified core genes (*JUN*, *IL6*, *TLR4*, *IFNG*) and critical pathways (PI3K-Akt, FoxO) represent reliable molecular candidates for the precise prevention and targeted treatment of BPA-related MASLD. Collectively, the present findings refine the theoretical basis for understanding environmental-mediated MASLD pathogenesis, underscore the importance of strengthening the regulatory control of BPA-relevant products, and provide innovative perspectives for the development of targeted MASLD intervention regimens. Future research will prioritize *in vitro* and *in vivo* functional validation of core molecular targets, exploration of subtype-dependent pathogenic differences, and toxicological assessment of mixed EDCs co-exposure, aiming to accelerate the translational transformation of basic research results into clinical practice and public health strategies.

## Data Availability

Publicly available datasets were analyzed in this study. This data can be found here: Gene Expression Omnibus, accession number https://www.ncbi.nlm.nih.gov/geo/query/acc.cgi?acc=GSE163211.
